# Coronary stent implantation links to the occurrence of eosinophilia and interstitial pneumonia: a case report and systematic review

**DOI:** 10.1186/s12890-024-03101-x

**Published:** 2024-06-17

**Authors:** Fuyun Zhang, Wei Wang, Yingwei Zhu, Yimin Mao, Tongsheng Wang, Pengfei Gao

**Affiliations:** Department of Respiratory and Critical Care Medicine, The First Affiliated Hospital, College of Clinical Medicine, Henan University of Science and Technology, Luoyang, Henan China

**Keywords:** Drug-eluting stents, Interstitial pneumonia, Eosinophil, Systematic review, Case report

## Abstract

**Background:**

Rapamycin has been extensively utilized for coating coronary artery stents to reduce the occurrence of restenosis, yet there has been limited research on the potential harms of rapamycin-eluting stents. Herein, We report a case of eosinophilia and interstitial pneumonia caused by a cobalt-based alloy stent eluted with rapamycin.

**Case presentation:**

The patient was admitted due to fever, cough, and expectoration symptoms. Previously, the patient had undergone a procedure of percutaneous coronary stent implantation in our hospital’s cardiology department, which led to a gradual rise in blood eosinophil count. This time, the eosinophil count was higher than the previous admission. A chest CT scan revealed multiple flocculent density increases in both lungs and bronchiectasis. The rapamycin-eluting stents may have caused eosinophilia and interstitial pneumonia, which improved after administering corticosteroids. A systematic review of relevant literature was conducted to summarize the characteristics of interstitial pneumonia caused by drug-eluting stents.

**Conclusion:**

Paclitaxel, everolimus, zotarolimus, and rapamycin are the types of drugs that can lead to drug-eluting stents, and because of the rarity of their onset, clinical doctors must be precise and prompt in diagnosing suspected cases to avoid misdiagnosis and delayed treatment.

## Background

Percutaneous coronary intervention is the primary treatment for coronary heart disease; however, the high restenosis rates with bare metal coronary stents have led to repeated revascularization surgeries. The introduction of drug-eluting stent technology has improved the safety and efficacy of this procedure [1]. However, there is still a risk of restenosis within the stent, and a few rare adverse reactions have occurred. This article reports a case of eosinophilia and interstitial pneumonia caused by rapamycin-eluting cobalt-based alloy stents and provides a comprehensive literature review to improve awareness of the potential adverse reactions of drug-eluting stents.

## Case presentation

The patient is a 75-year-old male who was admitted to the hospital on September 22, 2022, due to a month-long cough and sputum, which had been aggravated for the past three days. At the time of onset, the patient experienced fever, with the highest temperature reaching 38 °C and yellow pus-like sputum. He did not experience any chest tightness, chest pain, hemoptysis, dyspnea, rashes, or arthralgia. The fever subsided after receiving oral medication at a local clinic, but the cough and sputum did not improve. After receiving oral medication at a local town health center, the cough improved, and the sputum volume decreased, eventually turning into white, foamy sputum. The patient did not continue treatment. However, three days ago, the cough and sputum worsened, accompanied by wheezing at night, prompting him to seek treatment at the local county people’s hospital. A chest CT scan revealed bilateral interstitial pneumonia, with the right lung being the most prominent, and a blood routine showed a white blood cell count of 9.68 × 10^9^/L, neutrophils of 3.65 × 10^9^/L, and eosinophils of 3.65 × 10^9^/L, with a percentage of 37.7%. Subsequently, he was admitted for further diagnosis and treatment. Since the onset of the disease, the patient has been of clear consciousness, average mental state, slightly poor diet and sleep, and normal bowel movements, with no significant changes in weight. The patient’s medical history included radiofrequency ablation for paroxysmal supraventricular tachycardia 13 years ago, percutaneous coronary stent implantation for acute inferior myocardial infarction 11 months ago (2021.11.05), which was followed by long-term oral administration of “ticagrelor, indolbuprofen, and atorvastatin calcium tablets” (discontinued in March 2023). Nine months ago (2022.01.27), the patient was hospitalized in the gastroenterology department of our hospital due to “liver insufficiency” and reported that he had recovered but was not re-examined. Additionally, there were no notable abnormalities in the patient’s personal, family, marriage, childbirth, or vaccination histories.

### Admission physical examination

The body temperature was 36.9 ℃, the pulse was 82 beats/minute, the respiratory rate was 22 breaths/minute, and the blood pressure was 137/91 mmHg (1 mmHg = 0.133 kPa). The patient’s level of consciousness was lucid, and no cyanosis was present on the lips or nail beds. Upon examination of the pharyngeal cavity, there was no evidence of congestion or swelling in the bilateral tonsils. The thyroid gland was not enlarged. Furthermore, the jugular vein was not filled, and the hepatic jugular venous reflux sign was absent. The chest was symmetrical with no deformities, and symmetric respiratory movements were observed on both sides. Auscultation of the lungs revealed clear respiratory sounds with a few Velcro rales in the lower lungs. There was no protrusion in the precordial area, the boundary of cardiac dullness was not enlarged, the heart rate was 98 beats/minute, the heart rhythm was regular. No abnormal murmurs were heard in the auscultation area of each valve. The abdomen was soft to the touch, with no tenderness or rebound pain and no edema in the lower limbs.

### Laboratory examination

**Fig. 1:**
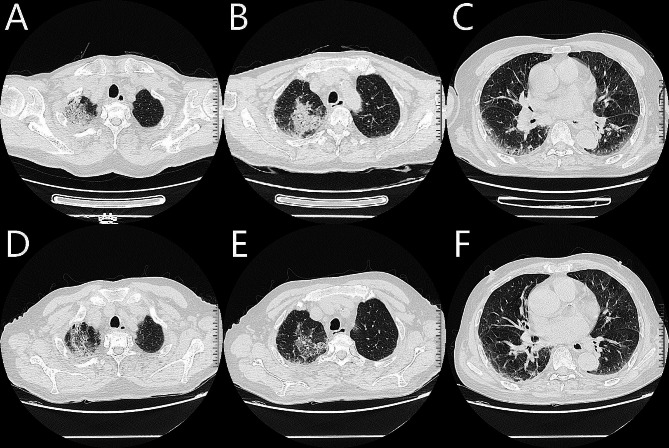
Comparison of chest HRCT scans before and after treatment. (**A-C**) Following admission on September 22, 2022, a chest HRCT revealed multiple flocculent density enhancement shadows in both lungs, with the right upper lobe being particularly prominent. (**D-F**) Following treatment with corticosteroids (methylprednisolone 40 mg, once every 12 h, for 3 days), the chest HRCT demonstrated a decrease in multiple flocculent density enhancement shadows in both lungs compared to the pre-treatment scan, as well as slight bronchiectasis in the apical segment of the right upper lobe

The blood routine examination revealed a white blood cell count of 9.30 × 10^9^/L, neutrophil count of 3.97 × 10^9^/L, eosinophil count of 3.66 × 10^9^/L, eosinophil percentage of 39.30%, hemoglobin of 112 g/L, and platelet count of 213 × 10^9^/L. The results of the urinary and fecal tests were normal. Blood biochemistry examination demonstrated albumin at 32.3 g/L, potassium at 3.43 mmol/L, and sodium at 124.6 mmol/L. The red blood cell sedimentation rate was measured at 85 mm/h. The thyroid function test revealed a free triiodothyronine level of 1.75 pg/mL, a free thyroxine level of 0.60 ng/dL, and a hypersensitive thyroid-stimulating hormone level of 44.97 uIU/mL. The antinuclear antibody test was positive at a fine particle type of 1:100, yet the autoantibodies for rheumatoid arthritis and vasculitis were negative. All other tests for liver and kidney functions, coagulation function, and infection-related pathogenic tests were normal. A chest CT scan revealed interstitial changes in the upper lobe of the right lung, with bipneumonic changes (Fig. 1. A-C). Additionally, a CT scan of the nasal sinuses showed evidence of complete sinusitis with deviated nasal septum. Pulmonary function testing demonstrated mild obstructive ventilatory dysfunction and mild pulmonary diffusion dysfunction. An electrocardiogram was performed and was found to be generally within normal limits. An echocardiogram revealed a widening of the ascending aorta, a lack of coordination in the basal segment of the left ventricular inferior wall, and decreased left ventricular diastolic function. Thyroid ultrasound showed bilateral lobular thyroid volume reduction and diffuse parenchymal lesions. To manage the symptoms, the patient was administered intravenous moxifloxacin (0.4 g, once daily) for anti-infection, cough relief, and phlegm reduction.

**Fig. 2:**
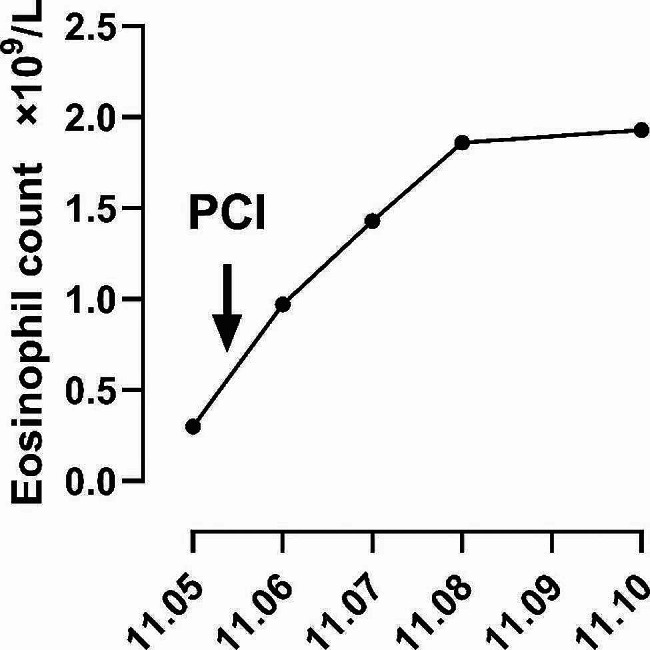
Changes of blood eosinophil levels post-stent implantation

By carefully examining the patient’s medical records from our hospital’s cardiology and digestive departments, we determined the patient’s basic eosinophil count in the blood. The patient was admitted to the cardiology department of our hospital on November 5, 2021, due to an acute inferior myocardial infarction, the urgent blood routine examination showed eosinophil count 0.30 × 10^9^/L. Subsequently, a rapamycin-eluting cobalt-based alloy stent (Firebird 2™) stent was implanted in the right coronary artery, resulting in a progressive increase in the eosinophil count, eosinophil counts were 0.97, 1.43, 1.86, and 1.93 × 10^9^/L on postoperative days 1, 2, 3, and 5, respectively (Fig. 2). On December 21, 2021, the outpatient department of cardiology in our hospital reviewed the blood routine and showed an eosinophil count of 1.83 × 10^9^/L. On January 28, 2022, the patient visited the digestive department of our hospital due to abnormally elevated liver enzymes, and two blood routine tests on admission still showed an increase in eosinophil count, which was 1.25 and 1.70 × 10^9^/L, respectively. Interstitial pneumonia was considered to be related to eosinophilia caused by coronary stent implantation, and the patient was administered methylprednisolone (40 mg, once/12 h, 3d; 40 mg, once/day, 3d). A chest CT scan showed a significant reduction in the lesion (Fig. 1. D-F). On February 2, 2022, the eosinophil count was 0.5 × 10^9^/L. It was advised to continue taking prednisone acetate orally outside the hospital (30 mg, once a day) and to return to the clinic after two weeks. However, due to family and financial reasons, the patient did not return to the clinic on time and stopped taking medication on their own. During a telephone follow-up on November 28, 2022, the patient was generally in good condition and did not report any coughing or expectoration.

### Systematic review

**Fig. 3:**
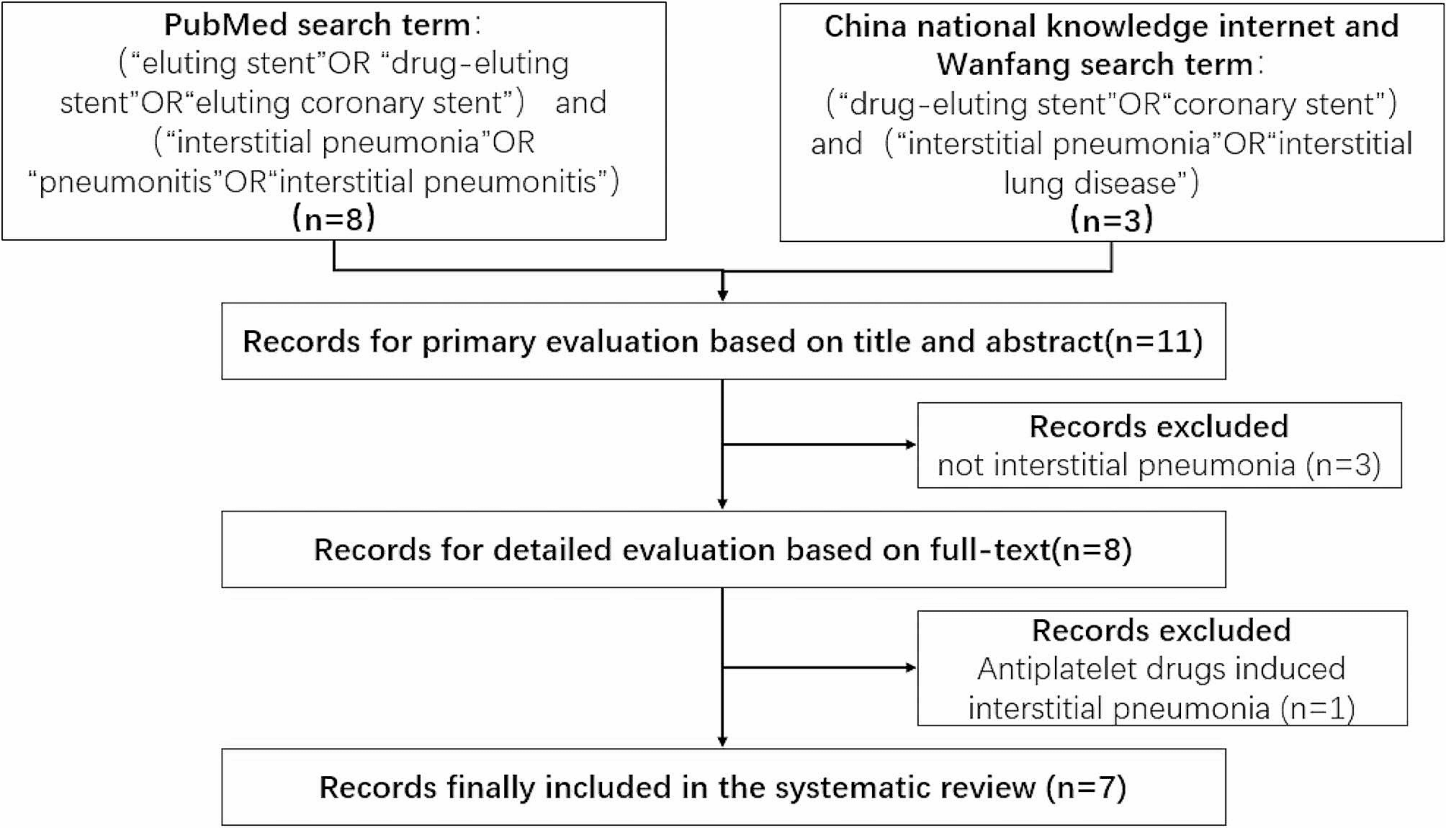
Flow diagram for literature review

We conducted a systematic literature search on November 10, 2023, without any language or publication year restrictions, using the terms (“eluting stent” OR “drug-eluting stent” OR “eluting coronary stent”) and (“interstitial pneumonia” OR “pneumonitis” OR “pneumoconiosis”) in the PubMed database, and (“drug-eluting stent” OR “coronary stent”) AND (“interstitial pneumonitis” OR “interstitial lung disease”) in China Wanfang and CNKI databases. After the literature search and the screening exclusion shown in Fig. 3, seven articles were included [2–8], comprising 5 in English, 1 in Chinese, and 1 in Japanese, involving eight patients.

This 75-year-old male patient experienced symptoms ten months after stent placement, which was a much longer delay compared to the eight patients discussed in the literature. His symptoms included fever, cough, expectoration, and high eosinophil levels in the blood. Imaging tests revealed multiple flocculent density increases in both lungs, which improved after corticosteroid treatment. This study included eight male patients, aged 54–76 years, with an average age of 68.25 years, who had drug-eluting stent-associated interstitial pneumonitis. The main symptoms reported were fever (7/8), dyspnea (6/8), cough (2/8), and chest tightness (1/8), with an average onset time of 32 days. The drugs associated with interstitial pneumonia were paclitaxel (3/8), everolimus (3/8), sirolimus (1/8), and zotarolimus (1/8). Only two cases showed elevated blood eosinophils. Chest CT scans revealed diffuse ground glass shadows in both lungs (6/8) and traction bronchiectasis or bronchiectasis with consolidation (3/8). Three cases of interstitial pneumonia caused by paclitaxel-eluting stent were diagnosed as acute interstitial pneumonia (AIP). One case of interstitial pneumonia caused by zotarolimus-eluting stent was identified as allergic pneumonia (HP). One interstitial pneumonia caused by everolimus-eluting stent showed infiltration of foam-like macrophages in the alveoli, with many inflammatory cells and lymphocytes in the interstitium. The remaining three cases had no pathological results. The remaining three cases had no pathological results. Apart from the three cases of interstitial pneumonia caused by paclitaxel-eluting stents, the other patients showed improvement after treatment with corticosteroids or long-term observation, with an overall mortality rate of 37.5%. However, the mortality rate of interstitial pneumonia caused by paclitaxel-eluting stents was 100%. The detailed characteristics of the included patients are presented in Table 1.

**Table 1:** Clinical characteristics of patients with interstitial pneumonia caused by drug-eluting stents

Author	Year	Age	Gender	Medicine	Onset time	Symptom	Eosinophils	Imaging examination	Pathologic examination	Treatment	Outcome
Kato K et al. [24]	2009	74	F	Paclitaxel	3 days	Fever, dyspnea	No increase	Traction bronchiectasis or bronchiolar dilatation with consolidation	Inflammatory cells were present in large numbers while eosinophils were present in fewer numbers in the pulmonary interstitial tissue, and diffuse involvement of the hyaline membrane	Corticosteroids (1 g/ day, 3 days); Minocycline; Meropenem	Died
Fujimaki T et al. [25]	2011	76	F	Paclitaxel	7 days	Fever, dyspnea	NA	Diffuse ground glass density and mild pleural effusion in both lungs	Diffuse alveolar injury with diffuse involvement of the hyaline membrane	Pulse steroid therapy for 3 days (dosage unknown)	Died
		74	F	Paclitaxel	3 days	Fever, dyspnea	NA	Diffuse ground glass density in both lungs	Diffuse alveolar injury with diffuse involvement of the hyaline membrane	Pulse steroid therapy(Dosage and duration unknown)	Died
Chiba T et al. [26]	2012	68	F	Everolimus	15 days	Fever, cough, and dyspnea	Increase (0.69 × 10^9^/L, 6.5%)	Diffuse ground glass density in both lungs	NA	Prednisolone(Started at 50 mg/ day and gradually decreased to 5 mg/ day after 2 months)	Improved
Sakamoto S et al. [27]	2013	72	F	Everolimus	1 month	Fever	No increase	Diffuse ground glass shadows in both lungs, tractive bronchiectasis or bronchiectasis with consolidation	Numerous foam macrophages, inflammatory cells, and lymphocytes were scattered amongst the interstitial tissue and a few eosinophils.	Prednisolone (Started at 30 mg/ day, gradually decreased to 5 mg/ day after 6 months)	Improved
Shin HW et al. [28]	2013	60	F	Zotarolimus	13 days	Fever, cough, and dyspnea	Increase (780/mm^3^)	Diffuse ground glass shadows and central lobular nodules	Chronic bronchiolitis was along with peribronchial fibrosis, inflammation, and luminal narrowing. A smattering of indistinct granulomas in the bronchiolar region featuring multinucleated giant cells.	High-dose steroids (2 weeks, dose unknown); Cefoxitin; roxithromycin	Improved
Jun Liu [29]	2018	54	F	Sirolimus	4 days	Fever, chest stuffiness	No increase	Thickened interstitial texture of outer lower lobe of both lungs, increased flake density, and uneven density	NA	Methylprednisolone (started at 80 mg/ day, gradually transitioned to 4 mg/ day); Imipenem; Oseltamivir	Improved
Kobayashi H [30]	2022	68	F	Everolimus	9 months	Dyspnea	NA	Mechanical pneumonia, including diffuse patchy air space consolidation, ground-glass shadows, and small nodular shadows	NA	No treatment	Improved

F, female; NA, not available

Onset time: The interval between the insertion of drug-eluting stents and the manifestation of interstitial pneumonia

## Discussion and conclusions

Elevation of eosinophils and interstitial pneumonia after drug-eluting stent implantation may be caused by three potential factors: antiplatelet drugs, intravenous injection drugs such as iodinated contrast agents, and the stent itself [9]. Oishi Y et al. reported a case of eosinophilic pneumonia that was attributed to the use of indomethacin suppositories. After ceasing the drug, the eosinophil count started to decrease and returned to a normal range within two months [10]. Despite the patient not having taken antiplatelet drugs for over 6 months prior to their hospitalization, the blood eosinophils were still high. It is unlikely that antiplatelet drugs were responsible for the increase in eosinophils, as the patient’s blood eosinophils remained elevated for a prolonged 10-month period following the insertion of the stent. This is in contrast to the iodinated contrast agent (iophedrolol injection 100 ml: 74.1 g, 2 doses) used during the procedure, which usually causes a spike in eosinophils that typically resolves within two days. Therefore, it is more likely that the drug-eluting stent itself was the culprit. The Firebird2TM rapamycin eluting cobalt-based alloy stent system utilized in this example is a second-generation drug-eluting stent independently developed and manufactured in China. The stent material is composed of L605 cobalt-based alloy, and the coating on the stent is a combination of rapamycin drug and a polymer biomaterial (styrene butene copolymer), with a drug content of 120–305 ug. Therefore, it is speculated that the patient’s eosinophil progression and interstitial pneumonia may have been caused by cobalt exposure or rapamycin.

Cobalt is a metal employed in various industrial processes, including the fabrication of hard metals. Unfortunately, exposure to cobalt can result in interstitial lung disease, although the precise cause of this is still unclear. This is because only a few exposed workers develop the disease, and there is no linear dose-response relationship, indicating that it may be linked to immunity [11]. The diagnosis of cobalt-related interstitial pneumonia is mainly determined by taking into account the patient’s occupational history, clinical symptoms, and radiological and pathological examination results. This condition is only observed in individuals exposed to cobalt through manufacturing and utilizing tools created from metal powders. Additionally, there have been a few reports of patients using e-cigarettes, as well as medical applications such as vascular stents and artificial joints [12, 13]. Wyles C C et al. concluded that when patients are implanted with artificial joints containing cobalt, the release of wear-related metals from the implant increases cobalt accumulation in myocardial tissue and a heightened frequency of pulmonary interstitial fibrosis [14]. In addition, those with vascular stents may be exposed to cobalt if the polymer coats rupture. Clinical signs of cobalt-related interstitial pneumonia can vary but most commonly include shortness of breath, coughing, fever, chills, and weight loss. High-resolution CT can be similar to non-specific interstitial pneumonia (NSIP), common interstitial pneumonia (UIP), sarcoidosis, or hypersensitivity pneumonitis (HP). The pulmonary alveolar lavage fluid can show an increase in the number of cells with a normal proportion or an increase in the number of lymphocytes, neutrophils, or eosinophils [15].

Giant cell interstitial pneumonia (GIP) is a common pathological manifestation of cobalt-associated interstitial pneumonia, characterized by the presence of central interstitial fibrosis in the bronchioles, accumulation of macrophages in the alveoli, and the presence of multinucleated macrophages with a “cannibalistic” nature. Increased eosinophils may also accompany it, although they are usually dispersed among macrophages and are not the main component [16]. In addition to GIP, cobalt-related ILD can be seen in histopathological manifestations such as HP, UIP, tissue pneumonia, and desquamated interstitial pneumonia (DIP) [12]. The patient’s symptoms in this case are consistent with the typical interstitial pneumonia caused by cobalt, and the HRCT findings are similar to those of HP. Previous research has demonstrated that alveolar lavage fluid from patients with cobalt-associated interstitial pneumonia can contain increased levels of eosinophils [15], and hypoxia-inducible factor 1 α Knockout mice also exhibited pulmonary eosinophil infiltration after acute cobalt injury [17]. However, no literature examines the relationship between cobalt and blood eosinophils. The patient’s eosinophil count increased post-stent implantation. However, there was no evidence of cobalt exposure from the stent’s polymer biomaterials. Therefore, the likelihood of cobalt-related interstitial pneumonia in this patient is low. Since the patient could not further examine the alveolar lavage fluid and lung tissue, the type of airway inflammation cannot be determined. Consequently, the diagnosis of cobalt-related interstitial pneumonia appears to be unfounded due to the absence of a clear exposure history or histopathological evidence.

Rapamycin, also known as sirolimus, was found in the soil from Easter Island in the early 1970s. This macrolide is derived from Streptomyces hygroscopicus [18]. Rapamycin has been proven to suppress the immune system and inhibit cell growth by targeting mammalian rapamycin targets (mTOR). Clinically, it has been utilized to prevent organ transplant rejection [19], lymphatic leiomyomatosis [20], and avert coronary artery stent restenosis. It has been difficult to accurately estimate the rate of interstitial pneumonia caused by rapamycin due to the absence of initial symptoms. A study on sirolimus-induced pneumonia in renal transplant patients revealed an incidence rate of 16.7%, with the average duration from oral medication to pneumonia being 14.7 ± 8.0 months [21]. The pathogenesis of this condition is yet to be fully understood, with potential causes being direct alveolar injury, immunogenic haptens, and immunologic drug reactions [22]. Clinically, it is characterized by bilateral interstitial infiltration, systemic symptoms, lymphocytic alveolitis (predominantly CD4 type), and may be associated with eosinophilia [23]. It has been previously documented that rapamycin can cause localized allergic contact dermatitis [24] and, when taken orally, can lead to lung hypersensitivity due to CD4 cell infiltration [25]. Thus, it is hypothesized that interstitial pneumonia might be an immune response rather than a result of dosage. The research regarding rapamycin and eosinophils is still contentious. A study conducted by HUA W et al. found that rapamycin significantly decreased the number of eosinophils in the local airway, peripheral blood, and bone marrow of a mouse model of OVA-induced allergic airway inflammation, regardless of IL-5 levels [26]. In contrast, Fredriksson K et al. found that rapamycin had a paradoxical effect on eosinophils when combined with exposure to house dust mite (HDM), resulting in a significant reduction of eosinophil counts in the alveolar lavage fluid (BALF). However, when rapamycin was used to treat asthma mice effectively induced by HDM, the amount of eosinophils in BALF increased. It is possible that there is a temporal connection between HDM stimulation and the mTOR pathway [27]. In the study by Mushaben EM et al., no significant increase in eosinophils was observed in BALF of HDM-induced asthma mice treated with rapamycin. However, the eosinophil levels remained at their original high levels, unlike when both HDM and rapamycin were administered simultaneously [28]. The concentration of rapamycin administered in the study was 1-4 mg/kg, which is much higher than the trace amount of rapamycin present in the stent, suggesting that the elevated eosinophils may be due to a hypersensitivity reaction rather than a therapeutic effect. In the literature review, only two patients were found to have elevated blood eosinophils, which may be attributed to the combination of other pathogenic microbial infections or a short time to implantation of drug-eluting stents.

The patient’s elevated eosinophil count may be indicative of stent stenosis. Studies have demonstrated that eosinophils accumulate in tissues near metal components of coronary artery stents, particularly polymer scaffolds [9]. Additionally, a previous study indicated that peripheral blood eosinophil count increases during restenosis [29]. Hajizadeh R et al. further established the predictive value of peripheral eosinophils for in-stent restenosis following coronary artery stent therapy for stable angina [30]. As the patient declined to undergo a post-treatment coronary angiography, the exact status of the stent remains unknown.

In conclusion, the patient’s eosinophilia and interstitial pneumonia are likely caused by rapamycin in coronary stents. Common clinical manifestations of interstitial pneumonia caused by drug-eluting stents include fever, dyspnea, and cough, which can be accompanied by diffuse ground glass shadows in both lungs, as observed in imaging. Early use of corticosteroids may be more beneficial, and a rise in eosinophil counts may suggest a positive response to the treatment. Therefore, it is essential to consider interstitial pneumonia caused by drug-eluting stent implantation in male patients with a recent history of the procedure, fever, and imaging showing diffuse ground glass shadows in both lungs, particularly in those who have had paclitaxel-eluting stent implantation. Since it is impossible to remove the 10-month-old rapamycin eluting stent, it is of utmost importance to make an accurate diagnosis of interstitial pneumonia and start glucocorticoid therapy as soon as possible.

## Data Availability

The data supporting this case report is available from the corresponding author on reasonable request.

## Abbreviations

AIP: Acute interstitial pneumonia
HP: Hypersensitivity pneumonia
HRCT: High-resolution computed tomography
NSIP: Non-specific interstitial pneumonia
UIP: Usual interstitial pneumonia
GIP: Giant-cell interstitial pneumonia
DIP: Desquamative interstitial pneumonia
mTOR: Mammalian target of rapamycin
OVA: Ovalbumin
IL-5: Interleukin-5
BALF: Bronchoalveolar lavage fluid
PCI: Percutaneous transluminal coronary intervention
